# Exosome-Mediated Delivery of Inducible miR-423-5p Enhances Resistance of MRC-5 Cells to Rabies Virus Infection

**DOI:** 10.3390/ijms20071537

**Published:** 2019-03-27

**Authors:** Jingyu Wang, Yawei Teng, Guanshu Zhao, Fang Li, Ali Hou, Bo Sun, Wei Kong, Feng Gao, Linjun Cai, Chunlai Jiang

**Affiliations:** 1 National Engineering Laboratory for AIDS Vaccine, School of Life Science, Jilin University, Changchun 130012, China; jingyu.wjy@foxmail.com (J.W.); tengyw17@mails.jlu.edu.cn (Y.T.); zhaoguanshu@foxmail.com (G.Z.); li296981502@126.com (F.L.); houal@jlu.edu.cn (A.H.); bo_sun@jlu.edu.cn (B.S.); weikong@jlu.edu.cn (W.K.); feng0215@gmail.com (F.G.); 2 Key Laboratory for Molecular Enzymology and Engineering of the Ministry of Education, School of Life Science, Jilin University, Changchun 130012, China

**Keywords:** host cell defense, microRNA, SOCS3, vaccine, rabies virus, miR-423-5p

## Abstract

The human diploid cell line Medical Research Council -5 (MRC-5) is commonly utilized for vaccine development. Although a rabies vaccine developed in cultured MRC-5 cells exists, the poor susceptibility of MRC-5 cells to the rabies virus (RABV) infection limits the potential yield of this vaccine. The underlying mechanism of MRC-5 cell resistance to RABV infection remains unknown. In this study, we demonstrate that viral infection increased exosomal release from MRC-5 cells; conversely, blocking exosome release promoted RABV infection in MRC-5 cells. Additionally, RABV infection up-regulated microRNA (miR)-423-5p expression in exosomes, resulting in feedback inhibition of RABV replication by abrogating the inhibitory effect of suppressor of cytokine signaling 3 (SOCS3) on type I interferon (IFN) signaling. Furthermore, intercellular delivery of miR-423-5p by exosomes inhibited RABV replication in MRC-5 cells. We also show that RABV infection increased IFN-β production in MRC-5 cells and that blocking the type I IFN receptor promoted RABV infection. In conclusion, MRC-5 cells were protected from RABV infection by the intercellular delivery of exosomal miR-423-5p and the up-regulation of IFN-β. These findings reveal novel antiviral mechanisms in MRC-5 cells against RABV infection. miR-423-5p, exosomes, and IFN signaling pathways may therefore be potential targets for improving MRC-5 cell-based rabies vaccine production.

## 1. Introduction

Rabies virus (RABV) is a negative-sense, non-segmented, single-stranded RNA virus that attacks the nervous systems of humans and other animals, causing rabies [1]. RABV infection caused approximately 17,400 deaths worldwide in 2015 and it remains a serious public health problem in many developing countries [2]. Prior to the development of the rabies vaccine by Louis Pasteur and Émile Roux in 1885, RABV infection in humans was typically fatal. Since the original nerve tissue-derived vaccine, the rabies vaccine has been under constant development, with the present vaccines being produced in cell cultures. However, development of a sufficiently effective, affordable, and high-quality rabies vaccine remains a major challenge in global rabies prevention and control [3]. In recent years, the potential use of cell culture techniques for vaccination against rabies and other viruses has been explored extensively. Cell culture-based rabies vaccines are more affordable and have proven to be safe and effective in preventing rabies infections in humans. Primary hamster kidney cells, bovine and dog kidney cells, purified chick embryo cells, purified duck embryo cells, fetal rhesus monkey lung cells, human diploid cells (HDCs), and Vero cells are widely used as substrates for rabies vaccine production [4,5,6,7]. Vaccines derived from HDCs are relatively safer than those derived from either explant animal primary cells or immortalized cells. Through adaptation and modification of the infection procedure, sustainable infections using several RABV strains in HDCs have been established. In 1974, HDC rabies vaccines (WI-38, MRC-5) were licensed and recommended by the World Health Organization as the gold standard [8]. However, MRC-5 cell expansion trains require long durations, cell culture procedures are complex, and cell yields are inefficient; vaccinia virus production is sub-optimal in MRC-5 cells owing to the inefficient cell yields. All these factors contribute to the prohibitively high cost of HDC-based rabies vaccine production, thus limiting its use in developing countries [8,9]. The human MRC-5 cell line is an intermediate susceptible cell line for RABV infection [10], indicating the presence of a resistance mechanism against RABV infection in MRC-5 cells. Elucidating this resistance mechanism would facilitate an increased capacity of MRC-5 cells for rabies vaccine production.

Diploid cells possess the capacity for self-defense—called cell-autonomous immunity [11]. A minimal set of germline-encoded antimicrobial defense factors enables an infected host cell to resist a variety of pathogens [12]. Type I interferons (IFN-α and IFN-β) are crucial participants in such cell-autonomous immunity. They are a group of signaling proteins synthesized and released by host cells and serve as powerful signals for eliciting the host defense in vertebrate cells [12]. Type I IFNs induce numerous gene families that encode effectors and regulators to direct protein antimicrobial or static activity. They can function as autocrine and paracrine factors, and create an antiviral state in nearby cells [13,14]. Because of this function, type I IFNs are successfully utilized as antiviral drugs in clinical practice. Although the Janus Kinase-Signal Transducer and Activator of Transcription (JAK-STAT) signaling pathway, mediated by IFNs, has been investigated extensively and substantial information is available, the detailed mechanisms underlying the regulation of this pathway are not fully characterized.

Exosomes, present in bodily fluids, are cell-derived bioactive membrane vesicles, with a typical diameter range from 30 to 100 nm [15]. They are generated and stored inside multivesicular bodies (MVBs), and are released into the extracellular environment upon MVB fusion with the plasma membrane [16]. Exosomes contain various ubiquitous and cell-specific molecules, including proteins, lipids, carbohydrates, and nucleic acids. They can discard undegraded endosomal or lysosomal proteins and membranes, suggesting waste management as the original function of exosomes [17]. In recent years, several studies have proved that exosomes may have other specialized functions, playing a crucial role in both physiological and pathophysiological processes such as alternative secretion of proteins, antigen presentation, intercellular signaling, RNA and infectious agent shuttling, and pathogen immune surveillance [18,19,20,21]. However, the exact biological functions of exosomes require further elucidation.

Recent studies suggest that the exosome is one of the key players in viral pathogenesis [22,23,24]. Exosomes released from virus-infected cells contain a variety of viral and host active molecules such as protein, lipids, full-length viral RNAs, and regulatory RNAs (e.g., miRNAs and small interfering RNAs) [21,25,26,27,28]. These exosomes have the ability to transfer the contained active molecules from one cell to another and thereby modify or reprogram target cells. Li et al. [29] reported that exosomes mediate the cell-to-cell transmission of IFN-α-induced antiviral activity. Giugliano et al. [30] showed that hepatitis C virus (HCV) infection can induce autocrine interferon signaling and exosomal release by human liver endothelial cells, thereby inhibiting viral replication. These studies indicate that the cell-to-cell transmission of viral resistance mediated by exosomes is a potential mechanism to amplify the interferon-induced antiviral response. However, the potential role of exosomes in MRC-5 cell self-defense against RABV infection remains unexplored.

In this study, we investigated whether exosomes participate in the resistance of MRC-5 cells against RABV infection and attempted to identify the antiviral molecule involved in this process. We found that blocking exosomal release promoted RABV infection in MRC-5 cells; conversely, exosomal microRNA (miR)-423-5p feedback inhibited RABV replication in MRC-5 cells by inhibiting the expression of suppressor of cytokine signaling 3 (*SOCS3*), a gene that encodes a STAT-induced STAT inhibitor (SSI). We prove that viral infection induced the exosomal cell-to-cell transfer of an antiviral microRNA that decreased rabies replication in MRC-5 cells.

## 2. Results

### 2.1. Blocking Exosomal Release Promotes RABV Infection in MRC-5 Cells

**Figure 1:**
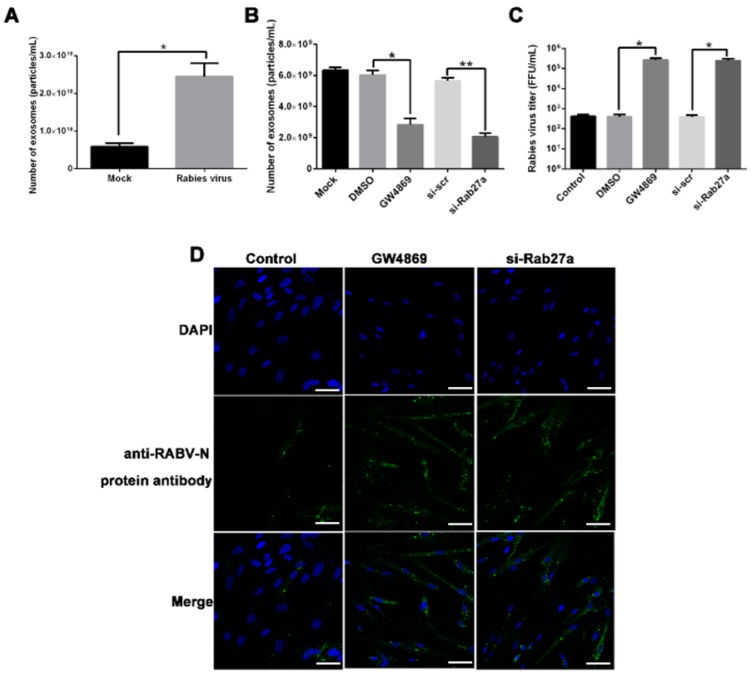
Blocking exosome release promotes rabies virus (RABV) infection in MRC-5 cells. (**A**) Quantification of exosomes from uninfected and RABV-infected MRC-5 cell culture supernatants (48 h) was performed using nanoparticle tracking analysis. (**B**) MRC-5 cells were treated with 10 µmol/L GW4869 for 6 h and transfected with 100 nmol/L si-Rab27A for 24 h; then, the culture medium was replaced with fresh media, and the cells were cultured for 48 h. Exosome concentration of the cell culture supernatant was confirmed by nanoparticle tracking analysis. (**C**) MRC-5 cells were treated with 10 µmol/L GW4869 for 6 h and transfected with 100 nmol/L si-Rab27A for 24 h, infected with (RABV; multiplicity of infection = 0.1) for 48 h. Then, the RABV titer of the cell culture supernatant was determined using quantitative reverse transcriptase PCR. (**D**) MRC-5 cells were treated and infected as described in (**C**). At 12 h post infection, cells were incubated with a fluorescein isothiocyanate-labeled antibody to the RABV N protein (green) for 2 h, then the cell nuclei were stained with 4′,6-diamidino-2-phenylindole (DAPI; blue). Scale bar = 50 µm.“Mock” and “Control” refer to uninfected cells and untreated RABV-infected cells, respectively. Three independent experiments were performed. * and ** indicate *p* < 0.05 and *p* < 0.01, respectively.

Recently, numerous studies have demonstrated that exosomes are involved in many processes related to viral infection. We previously reported that RABV infection in susceptible Vero cells promoted the release of exosomes and that exosomes potentially facilitated viral infection. However, the role of exosomes in RABV infection in MRC-5 cells, a non-susceptible cell line, was heretofore unknown. In the present study, we found that the number of exosomes in the culture supernatant of RABV-infected MRC-5 cells was significantly higher (*p* < 0.05) than that in uninfected cells (Figure 1A). Next, treatment with two inhibitors of exosome release, GW4869 and si-Rab27A, and subsequent nanoparticle tracking analysis revealed that the number of exosomes released from MRC-5 cells was significantly lower following GW4869 (*p* < 0.05) or si-Rab27A (*p* < 0.01) treatment (Figure 1B). Additionally, the inhibitor treatments significantly increased viral tilters in the culture supernatants (*p* < 0.05; Figure 1C). Confocal microscopy confirmed that GW4869 and si-Rab27A treatments promoted RABV infection in MRC-5 cells (Figure 1D). These data suggest that RABV infection enhanced exosome release, which in turn caused feedback inhibition to impair further RABV infection in MRC-5 cells.

### 2.2. RABV Infection Alters miRNA Expression Patterns in Exosomes

**Figure 2:**
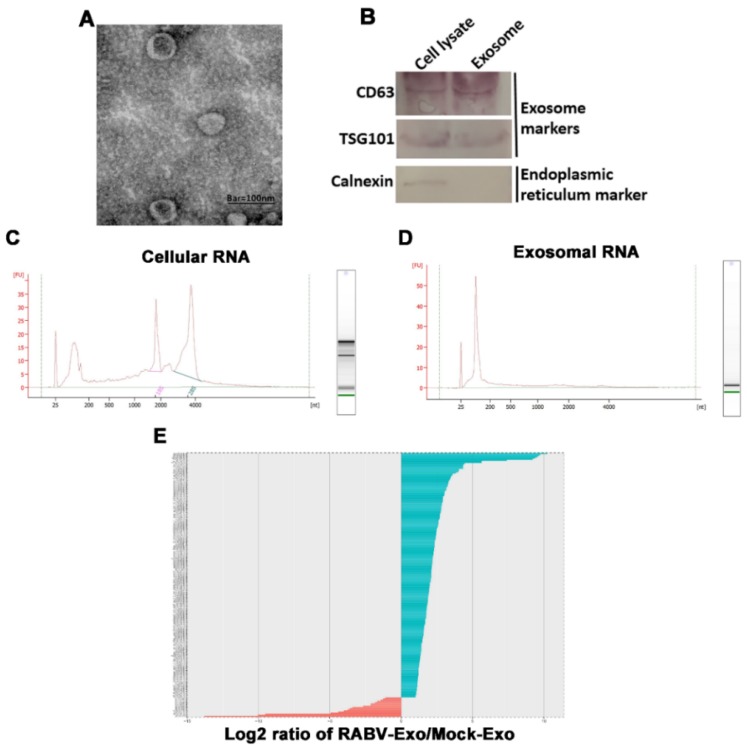
Characterization of exosomes and exosomal small RNA deep sequencing. (**A**) Exosomes from rabies virus (RABV)-infected MRC-5 cell culture supernatants were negatively stained with 2% phosphotungstic acid and analyzed using transmission electron microscopy. Scale bar = 100 nm. (**B**) Western blotting analysis of the isolated exosomes using the exosome-specific markers CD63 and TSG101 and the non-exosomal marker calnexin. Total RNA from RABV-infected MRC-5 cells (**C**) and exosomes released from RABV-infected MRC-5 cells (**D**) were detected using an Agilent 2100 bioanalyzer. The corresponding virtual gel images generated using the software are depicted as electropherograms. (**E**) Distribution of 232 microRNAs (miRNAs) were differentially expressed in exosomes isolated from RABV-infected and uninfected cells. A total of 215 miRNAs were detected as up-regulated (log2 > 1) and 17 miRNAs were detected as down-regulated (log2 < 1).

Previous studies have shown that miRNAs in exosomes are involved in the host defense against viral infection. Here, we performed the deep sequencing of exosomal miRNAs isolated from the culture supernatants of uninfected (Mock-Exo) and RABV-infected (RABV-Exo) MRC-5 cells and analyzed the expression patterns. First, we isolated and purified exosomes using ultracentrifugation, and then identified and characterized the exosomes using electron microscopy and western blotting. Transmission electron microscopy (TEM) data indicated that the isolated particles had morphologies typical of exosomes (Figure 2A). The exosome fraction had observable signal for the exosome-specific markers CD63 and TSG101, but no observable signal for the endoplasmic reticulum marker calnexin (Figure 2B). These data demonstrate that the methods described here can be used to isolate exosomes from the culture supernatants of RABV-infected cells.

Next, the total RNA was analyzed by deep sequencing. Figure 2C shows that the RNA of virus-infected MRC-5 cells mainly consisted of 18S and 28S ribosomal RNA (rRNA) and some small RNAs. However, RABV-Exo samples contained abundant small RNAs (19–22 nucleotides) but little to no trace of 18S or 28S ribosomal RNAs (Figure 2D). In this study, 232 miRNAs in total (215 miRNAs up-regulated and 17 miRNAs down-regulated) were identified using miRNA deep sequencing as differentially expressed between RABV-Exo and Mock-Exo samples (Figure 2E, Table S1). Taken together, these results indicate that the RNAs within exosomes released from MRC-5 cells mainly consisted of miRNAs and that RABV infection altered the expression profile of miRNAs contained in exosomes.

### 2.3. miR-423-5p Targets Suppressor of Cytokine Signaling 3 (SOCS3)

**Figure 3:**
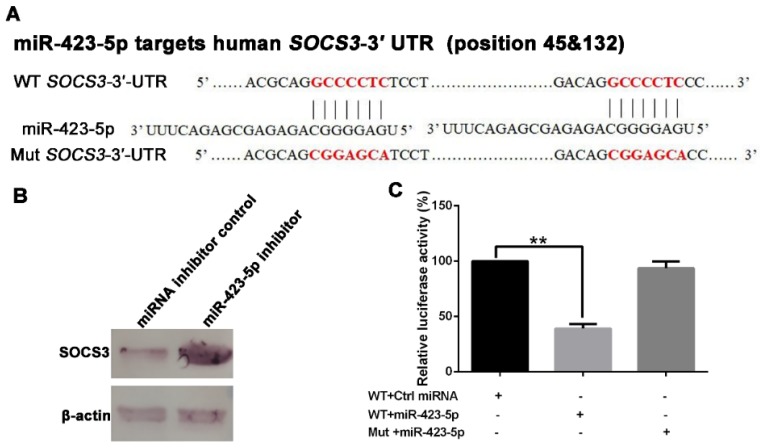
MicroRNA (miR)-423-5p targets the suppressor of cytokine signaling 3 (*SOCS3*) gene. (**A**) Schematic of miR-423-5p target region of *SOCS3*. The RNA sequences of the miR-423-5p wild type (WT) and mutant (Mut) forms are shown. (**B**) MRC-5 cells were transfected with an miRNA-423-5p inhibitor or negative miRNA inhibitor control for 12 h, the culture medium was replaced with fresh media, and the cells were cultured for 48 h. The cells were harvested and the levels of SOCS3 were determined using western blotting. (**C**) MRC-5 cells were co-transfected with the wild-type *SOCS3* 3′-UTR luciferase vector, mutant *SOCS3*-3′ UTR, and 100 nmol/L miRNA-423-5p mimic. Relative luciferase activity was measured after 48 h of transfection. The control miRNA mimic was also used as a negative control. Three independent experiments were performed. ** indicates *p* < 0.01.

According to the bioinformatics analysis of the differentially expressed exosomal miRNAs, performed with TargetScan and miRanda, the significantly up-regulated miR-423-5p, miR-493-3p, and miR-383-5p were predicted to target the 3′-untranslated region (UTR) of *SOCS3*. A schematic of the miR-423-5p target sites within the 3′-UTR of *SOCS3* is shown in Figure 3A. Western blotting was used to determine whether the level of SOCS3 in MRC-5 cells was targeted by miR-432-5p, miR-493-3p, or miR-383-5p. We transfected 100 nmol/L each of miRNA-423-5p inhibitor, miR-493-3p inhibitor, miR-383-5p inhibitor, or negative miRNA inhibitor control into MRC-5 cells; western blotting demonstrated that SOCS3 expression increased in the presence of miRNA-423-5p inhibitor (Figure 3B). However, neither the miR-493-3p inhibitor nor the miR-383-5p inhibitor affected SOCS3 expression (Figure S1). Furthermore, dual-luciferase reporter assays were used to confirm the interaction between the *SOCS3* 3′-UTR and miR-423-5p. As shown in Figure 3C, luciferase activity significantly decreased in MRC-5 cells co-transfected with miR-423-5p mimic and the wild-type SOCS3-3′-UTR (WT-*SOCS3*-3′-UTR), but not in cells co-transfected with miR-423-5p mimic and a mutant *SOCS3*-3′-UTR (Mut-*SOCS3*-3′-UTR; Figure 3C). These data suggest that miR-423-5p targets the *SOCS3* 3′-UTR and thereby inhibited the expression of SOCS3.

### 2.4. miR-423-5p Feedback Inhibits RABV Replication in MRC-5 Cells

**Figure 4:**
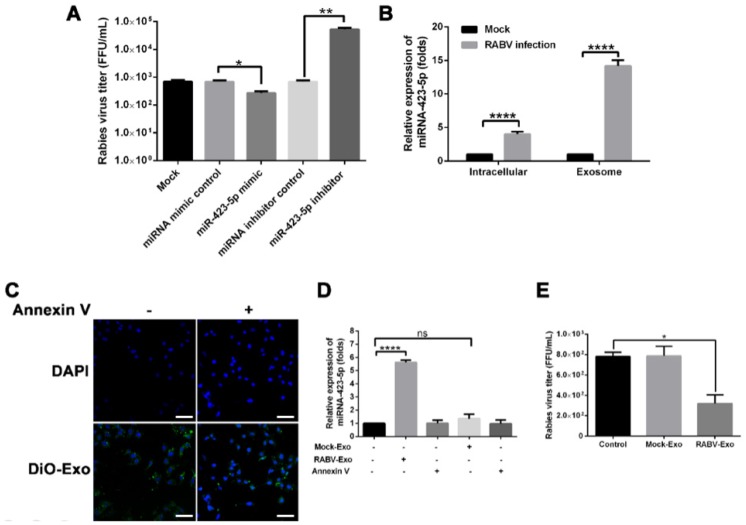
MicroRNA (miR)-423-5p feedback inhibits rabies virus (RABV) replication in MRC-5 cells. (**A**) MRC-5 cells were transfected with 100 nmol/L miR-423-5p mimic, miRNA mimic control, miR-423-5p inhibitor, or miRNA inhibitor control for 24 h, and the culture medium was replaced by fresh media containing RABV (multiplicity of infection (MOI) = 1). At 48 h post infection, the viral titer was determined using quantitative reverse transcriptase PCR (qRT-PCR). (**B**) qRT-PCR analysis of miR-423-5p expression in intracellular compartments or purified exosomes of MRC-5 cells with or without RABV infection for 24 h. (**C**) Exosomes from RABV-infected MRC-5 cell culture supernatants (RABV-Exo) were labeled with DiO dye, incubated with MRC-5 cells for 20 min, and visualized using fluorescence confocal microscopy. The annexin V-treated groups were pre-incubated for 2 h with annexin V (2 µg/mL). Scale bar = 50 µm. (**D**) qRT-PCR analysis of miR-423-5p levels in the recipient MRC-5 cells. Uninfected MRC-5 cells were incubated with purified exosomes from uninfected MRC-5 cells (Mock-Exo) or RABV-infected cells (RABV-Exo) in the presence or absence of annexin V. At 24 h post incubation, the miR-423-5p expression levels were determined. (**E**) MRC-5 cells were pre-incubated with RABV-Exo or Mock-Exo for 12 h, infected with RABV (MOI = 1) for 48 h, and the viral titers were determined using qRT-PCR. Three independent experiments were performed. *, **, and **** indicate *p* < 0.05, *p* < 0.01, and *p* < 0.0001, respectively.

To explore the biological function of miR-423-5p during RABV infection, the effect of miR-423-5p on RABV replication in MRC-5 cells was examined. We found that the miR-423-5p mimic suppressed RABV replication, while the miR-423-5p inhibitor facilitated RABV replication in MRC-5 cells (Figure 4A). RABV infection enhanced the release of exosomes; conversely, reduced exosome release promoted RABV infection (Figure 1A,C,D). Furthermore, qRT-PCR assays showed that the miR-423-5p levels were significantly higher in exosomes isolated from RABV-infected cells than those from the intracellular fraction or from uninfected cells (Figure 4B). Based on these findings, we further examined the effect of exosomal miR-423-5p on RABV infection in MRC-5 cells. First, we evaluated whether exosomes could transfer miR-423-5p to target cells. We labeled isolated exosomes with the green fluorescent lipid dye DiO, incubated them with MRC-5 cells for 24 h, and visualized them using confocal microscopy. MRC-5 cells efficiently internalized DiO-labeled exosomes; however, pre-incubation with annexin V, which binds phosphatidylserine at the exosome surface and thereby inhibits exosome uptake into cells, diminished DiO-labeled exosome internalization (Figure 4C). Incubation of MRC-5 cells with RABV-Exo significantly increased the levels of miR-423-5p in recipient cells, whereas incubation with Mock-Exo had no effect on the expression level of miR-423-5p (Figure 4D). However, when cells were pre-incubated with annexin V, thereby blocking exosome uptake into cells, the increase in miR-423-5p expression was eliminated. These data indicate that exosomes isolated from RABV-infected cells could in turn deliver miR-423-5p to MRC-5 cells. We also examined the effect exosomal miR-423-5p on RABV replication in MRC-5 cells. MRC-5 cells were pre-incubated with RABV-Exo or Mock-Exo, incubated with RABV (multiplicity of infection (MOI) = 1) for 48 h, and the viral titers were determined using qRT-PCR. As shown in Figure 4E, the viral titers of MRC-5 cells pre-incubated with RABV-Exo were significantly lower than those pre-incubated with Mock-Exo or the control groups. These results imply that exosomal miR-423-5p could inhibit RABV replication in MRC-5 cells.

### 2.5. RABV Infection Induces Up-Regulation of IFN-β in MRC-5 Cells

**Figure 5:**
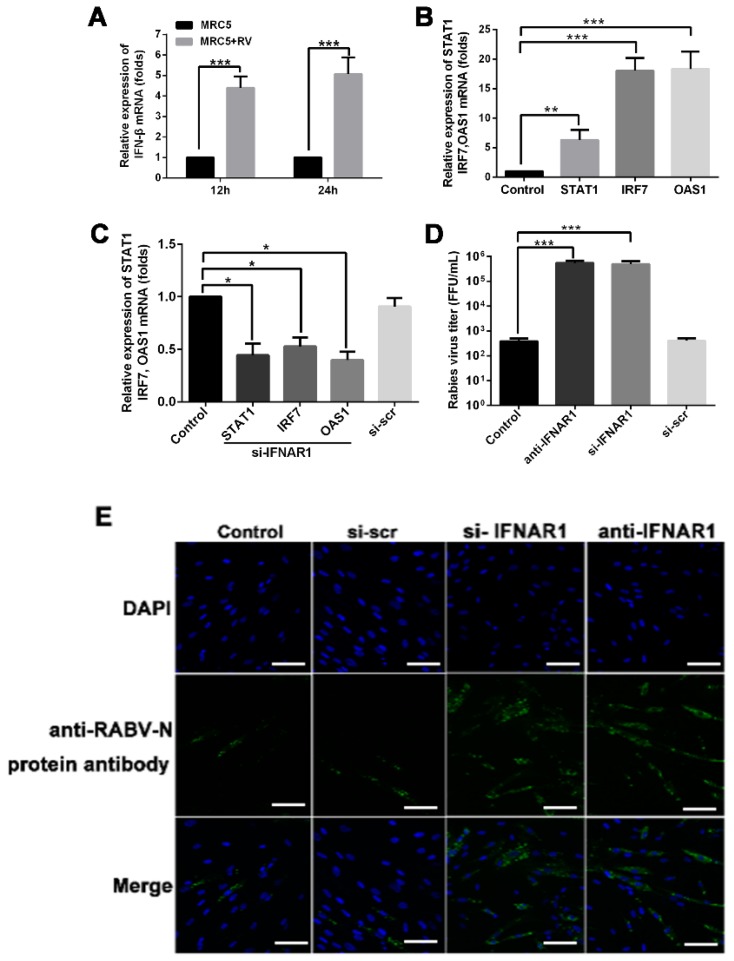
Rabies virus (RABV) infection induces up-regulation of interferon (IFN)-β in MRC-5 cells. (**A**) MRC-5 cells were infected with RABV (multiplicity of infection (MOI) = 0.1) for 12 h or 24 h. IFN-β expression was measured by quantitative reverse transcriptase PCR (qRT-PCR). (**B**) MRC-5 cells were infected with RABV (MOI = 0.1) for 24 h, and the expression levels of *STAT1*, *IRF7*, and *OAS1* were determined by qRT-PCR. (**C**) MRC-5 cells were transfected with 100 nmol/L an siRNA targeting *IFNAR1* or an siRNA negative control (si-scr), and infected with RABV at 24 h post transfection. Total RNA was extracted 24 h post infection, and the expression levels of the mRNAs encoding the JAK-STAT pathway proteins of STAT1, IRF7, and OAS1 were determined using qRT-PCR. (**D**) MRC-5 cells were treated with 100 nmol/L of an siRNA targeting *IFNAR1* (si-IFNAR1) for 24 h or 10 µg/mL of an anti-IFNAR monoclonal antibody for 6 h. After the change of culture media, the MRC-5 cells were infected with RABV (MOI = 0.1). At 48 h post infection, the RABV titers in cell supernatants were determined using qRT-PCR. (**E**) MRC-5 cells were treated with 100 nmol/L of an siRNA targeting *IFNAR1* (si-IFNAR1) for 24 h or 10 µg/mL of an anti-IFNAR monoclonal antibody for 6 h. After the change of culture media, MRC-5 cells were infected with RABV (MOI = 0.1). At 12 h post infection, the cells were incubated with fluorescein isothiocyanate-labeled RABV N protein antibody (green) for 2 h, and the cell nuclei were stained with 4′,6-diamidino-2-phenylindole (blue). Scale bar = 50 µm. Three independent experiments were performed. *, **, and *** indicate *p* < 0.05, *p* < 0.01, and *p* < 0.001, respectively.

Classical type I IFN signaling activates the JAK-STAT pathway, leading to transcription of IFN-stimulated genes. The production of type I interferons (IFN-α and IFN-β) by host cells is a primary response against viral infection. Therefore, we also explored the expression profile of type I IFNs in MRC-5 cells using qRT-PCR. We found that the levels of the mRNA encoding IFN-β were significantly higher in RABV-infected MRC-5 cells than those in untreated MRC-5 cells (Figure 5A). The levels of genes related to the IFN-β-associated JAK-STAT signaling pathway—*STAT1*, *IRF7*, and *OAS1*—were also up-regulated in RABV-infected MRC-5 cells (Figure 5B). Virus-infected MRC-5 cells treated with 100 nmol/L of an siRNA against *IFNAR1* (si-IFNAR1) to block its expression exhibited down-regulated expression of *STAT1*, *IRF7*, and *OAS1* (Figure 5C). Furthermore, blocking IFNAR1 action using a targeted siRNA and anti-IFNAR1 monoclonal antibodies significantly increased the level of viral RNA in the culture supernatant relative to that in control groups (Figure 5D). Consistent with this finding, the confocal microscopy data indicate that MRC-5 cells treated with si-IFNAR1 or anti-IFNAR1 monoclonal antibody were susceptible to RABV infection (Figure 5E). These data indicate that IFN-β plays an important role in MRC-5 cell defense against infection.

## 3. Discussion

In this study, we showed that RABV infection increased IFN-β expression and induced the transfer of antiviral miRNAs from RABV-infected MRC-5 cells to neighboring cells via exosomes. We demonstrated that the inducible antiviral miRNA miR-423-5p targeted *SOCS3* and might abrogate the inhibitory effect of SOCS3 on the IFN signaling pathway. Additionally, the increased expression of IFN-β is defense mechanism of MRC-5 cells against RABV infection. Thus, the exosomal miR-423-5p and the up-regulated IFN-β expression might enhance the antiviral effects of IFN signaling in the resistance of RABV replication in MRC-5 cells. These findings suggest novel mechanisms of the host cell defense against RABV infection in MRC-5 cells and provide insights to support further studies for improvement of MRC-5 cell-based rabies vaccine production.

The cell-autonomous immunity against viral infection involves the up-regulated expression of cellular antiviral molecules that are mediated by the type I IFN signaling pathways [11,12]. In response, viruses utilize several strategies to interfere with IFN signaling and evade the host antiviral immune responses [14]. However, the IFN-induced antiviral response is still able to control most viral infections, and it is indispensable for controlling viral infections in vertebrates [14]. In this study, we demonstrated that RABV infection increased the expression of IFN-β and the IFN signaling-associated genes in MRC-5 cells (Figure 5A,B). Blocking IFNAR1 enhanced RABV infection (Figure 5D,E). Following incubation with the RABV challenge virus standard (CVS) strain (MOI = 1), the limited susceptibility of the MRC-5 cell line results in only approximately 30% of cells being infected after 24 h [10]. We found that the MRC-5 cell line was not susceptible to infection with the RABV PM1503 strain (Figure S2). Our data confirmed that IFN-β signaling played an important role in the cell-autonomous immune response of MRC-5 cells against RABV infection. Numerous studies have demonstrated that exosomes participate in cell-to-cell communication and the transfer of biologically active molecules [21,26]. Recent studies have shown that exosomes released from cells infected with viruses, such as human immunodeficiency virus, human T-cell lymphotropic virus, HCV, Epstein-Barr virus (EBV), herpesvirus, or human papillomavirus, harbor and deliver many biologically active molecules, including viral and cellular RNAs and proteins, to neighboring cells, helping to establish productive infections [22,23,31,32,33,34]. On the other hand, additional studies have demonstrated that exosomes mediate intercellular transmission of antiviral molecules (e.g., type I IFNs, NKG2D ligands, and miRNA-29) and limit viral infection [29,30,35,36]. These studies suggest that exosomes can either spread or limit a viral infection depending on both pathogen identity and target cell type. Until now, the function of exosomes in the process of RABV infection in MRC-5 cells was unknown. Our study shows that RABV infection increased the release of exosomes into cell culture supernatants (Figure 1A). Additionally, blocking the exosome release enhanced the viral infection in MRC-5 cells (Figure 1C, D). These data imply that exosomal feedback induced by RABV infection impaired viral infection in MRC-5 cells.

Next, we focused on the mechanism of exosome-mediated antiviral effects. First, we found that miRNAs are abundant in exosomes (Figure 2D) and that RABV infection alters exosomal miRNA expression patterns (Figure 2E and Table S1). These findings are consistent with previous studies [28,33,37,38,39]. Chugh et al. reported that exosomes derived from Kaposi’s sarcoma-associated herpesvirus (KSHV) contain circulating miRNAs and that oncogenic and viral miRNAs could mediate KSHV pathogenesis [40]. Ariza et al. reported that EBV miRNAs are transferred by exosomes, a possible mechanism of intercellular communication and immune regulation [32]. Among the up-regulated miRNAs in RABV-infected exosomes, miR-423-5p was identified as related to the *SOCS3* gene (Figure 3). In humans, the *SOCS3* gene encodes the SOCS3 protein, a member of the SOCS family, also known as the SSI family that operates as a part of the negative feedback system of the JAK-STAT pathway [41]. SOCS3 can impair type I IFN signaling by regulating the strength and duration of STAT signaling [41]. Recent studies have demonstrated that miR-423-5p plays a pivotal role in modulating numerous cellular activities [42,43]. However, only a few studies have reported on the regulation of type I IFN signaling by miRNAs. To better understand the underlying role of miR-423-5p against RABV infection in MRC-5 cells, we characterized the biological function of miR-423-5p. Overexpression of miR-423-5p inhibited *SOCS3* gene expression in MRC-5 cells (Figure 3C). A miR-423-5p inhibitor facilitated RABV replication in MRC-5 cells, whereas additional miR-423-5p mimic suppressed RABV replication (Figure 4A). Additionally, exogenous exosomes were efficiently internalized by MRC-5 cells (Figure 4C), and the exosomes were able to deliver miR-423-5p to the recipient cells (Figure 4D). Furthermore, exosomal miR-423-5p inhibited RABV replication in MRC-5 cells (Figure 4E). Collectively, these data clearly demonstrate that exosomes can deliver miR-423-5p from RABV-infected MRC-5 cells to neighboring cells and that miR-423-5p can abrogate the inhibitory effects of SOCS3 on the IFN signaling pathway by targeting the *SOCS3* gene, thus resulting in cell-autonomous immunity of MRC-5 cells to RABV infection. However, we did not exclude the possibility that endogenous miR-423-5p or other induced miRNAs could also influence the MRC-5 cell-autonomous immune response against RABV infection.

There are several potential limitations to this study. First, although we demonstrate that both the enhanced expression of type I IFN and exosomal miR-423-5p play important roles in resisting RABV replication in MRC-5 cells, the detailed molecular mechanism underlying IFN signaling pathway remains to be defined. Second, whether the inducible exosome-mediated delivery of miR-423-5p to induce antiviral activity is a universal mechanism in MRC-5 cells for controlling infection with different RABV strains remains unknown. Third, we did not exclude the possibility that exosomes mediate the cell-to-cell transmission of IFN-β-induced antiviral activity in MRC-5 cells.

In summary, we show that RABV-infected MRC-5 cells initiated the inducible expression of exosomal miR-423-5p, and thereby promoted a type I IFN response targeting *SOCS3*. We also show that RABV infection increased the production of antiviral cytokine IFN-β in MRC-5 cells. These findings suggest novel antiviral mechanisms in MRC-5 cells against RABV infection. Hence, miR-423-5p, exosomes, and the type I IFN signaling pathway may be potential targets for improving MRC-5 cell-based rabies vaccine production, and therefore represent promising therapeutic topics before further investigation.

## 4. Materials and Methods

### 4.1. Cell Culture

Human embryonic lung fibroblast cells (MRC-5) obtained from American Type Culture Collection (Manassas, VA, USA; CCL-171) were cultured in Minimum Essential Medium (Invitrogen, Carlsbad, CA, USA) containing 10% exosome-free fetal bovine serum, 100 IU/mL penicillin, and 100 mg/mL streptomycin at 37 °C in an atmosphere containing 5% CO_2_.

### 4.2. Exosome Isolation

Exosomes from cell supernatants were purified by differential centrifugation, as described previously [44]. In brief, MRC-5 cells were mock infected or infected with RABV (PM 1503, MOI = 0.1). Twelve hours post infection, the cell supernatants were collected and centrifuged at 300× *g* for 10 min, 2000× *g* for 10 min, 10,000× *g* for 30 min, and 100,000× *g* for 90 min. The obtained exosome-containing pellets were washed once with phosphate-buffered saline (PBS) and centrifuged again at 100,000× *g* for 90 min. Purified exosomes were then analyzed using TEM, nanoparticle tracking analysis (NTA), and western blotting. The RABV strain PM 1503 was propagated in Vero cells, and 72 h post infection, the cell culture supernatants were harvested, purified, and stored at −80 °C.

### 4.3. Exosome Labeling and Uptake Analysis

For exosome uptake experiments, exosomes were isolated from RABV-infected MRC-5 cells and then were labeled with the carbocyanine dye dioctadecyloxacarbocyanine perchlorate (DiO; Biotium, Inc., Fremont, CA, USA), as described previously [45]. In brief, exosomes diluted in PBS were incubated in 5 µmol/L DiO dye for 20 min, the labeled exosomes were washed at 100,000× *g* for 1 h to remove the excess dye, and the exosome pellet was diluted to 100 μL in PBS and added to recipient MRC-5 cells for uptake experiments. For exosome internalization experiments, annexin V was used, as it has been reported to block phosphatidylserine at the exosome surface and thereby inhibit exosome entry into cells [19,29]. Recipient MRC-5 cells were pre-incubated with 2 μg/mL annexin V (BD Biosciences, San Jose, CA, USA), incubated with DiO-labeled exosomes for 24 h, and analyzed using fluorescence microscopy using the Zeiss LSM 710 (Carl Zeiss, Oberkochen, Germany).

### 4.4. Small RNA Deep Sequencing Analysis

Exosome samples were isolated from RABV-infected and uninfected cells, as described above. Total RNA samples were isolated from exosomes using TRIzol reagent (Invitrogen, Carlsbad, CA, USA), according to the manufacturer’s directions. The total RNA concentrations were determined using a NanoDrop spectrophotometer (Thermo Fisher Scientific, Waltham, MA, USA), and RNA quality was analyzed using a 2100 Bioanalyzer (Agilent Technologies, Santa Clara, CA, USA). Deep sequencing of three separate exosomal preparations was performed by BGI (Shenzhen, China) with the BGISEQ-500 platform to determine their miRNA profiles. Bioinformatics analysis were performed by using TargetScan (http://www.targetscan.org) and miRanda (http://www.microna.org/microrna/home.do)

### 4.5. Transient Transfection of siRNAs and miRNAs

The miRNAs and siRNAs were transfected into 30–50% confluent MRC-5 cells using Lipofectamine^TM^ 2000 (Invitrogen), according to the manufacturer’s protocol. The final concentrations of si-IFNAR1, si-Rab27A, miR-423-5p mimic, miRNA mimic control, miR-423-5p inhibitor, miR-493-3p inhibitor, miR-383-5p inhibitor, and miRNA mimic control (all from RiboBio Biotech, Guangzhou, China) were 100 nmol/L each. The effect of transfection was detected using qRT-PCR and western blotting. The MRC-5 cells transfected with 100 nmol/L si-IFNAR1 and si-Rab27A for 12 h; then, the culture media was replaced with fresh media, the cells were cultured for 48 h, and qRT-PCR was performed. The MRC5 cells were transfected with miRNA-423-5p mimic, miRNA mimic control, miR-423-5p inhibitor, miR-493-3p inhibitor, miR-383-5p inhibitor, and miRNA mimic control for 12 h, the culture media was replaced with fresh media, and the cells were cultured for 48 h. SOCS3 protein levels were detected to analyze the effect of miRNA targeting SOCS3 levels.

### 4.6. Luciferase Reporter Assays

Wild-type (WT) and mutant (Mut) *SOCS3* mRNA 3′-UTR constructs were inserted into a luciferase reporter plasmid (Biobio Biotech). MRC-5 cells were co-transfected with the WT or Mut *SOCS3* mRNA 3′-UTR luciferase reporter plasmid and 100 nmol/L miR-432-5p mimic or miRNA mimic control. After transfection for 48 h, the cells were lysed and the relative luciferase activity was determined using the Dual-Luciferase^®^ Reporter Assay System (Promega Corporation, Madison, WI, USA). Firefly luciferase activity was normalized to *Renilla* luciferase activity.

### 4.7. RNA Extraction and Quantitative RT-PCR

Total RNA was extracted using the TRIzol reagent (Invitrogen). One Step PrimeScript™ RT-PCR Kit II (Takara Bio, Inc., Kusatsu, Japan) and miRNA two-step qRT-PCR (TransGen Biotech Co., Ltd., Beijing, China) were used with the CFX96 system (Bio-Rad Laboratories, Inc., Hercules, CA, USA) to quantify RABV, IFN-β, STAT1, IRF7, OAS1, and 18S rRNA. 18S rRNA was used for mRNA normalization. Quantification of miR-423-5p was performed using miR-423-5p-specific forward primers and universal reverse primers. Exogenous *Caenorhabditis elegans* miR-39 (cel-miR-39) was used for microRNA normalization. All reactions were performed in triplicate and analyzed using the 2^−ΔΔ*C*t^ method. The sequences of the qRT-PCR primers used were as follows (Table 1) and have been described previously [35,46,47].

### 4.8. Western Blotting

Cells and exosome samples were lysed in radioimmunoprecipitation assay buffer and boiled for 10 min. Lysate proteins were separated on a 12% polyacrylamide gel by electrophoresis. The proteins were transferred onto polyvinylidene difluoride membranes (EMD Millipore, Burlington, MA, USA). The membranes were blocked in 3% non-fat dry milk for 1 h at room temperature (approximately 25 °C) and subsequently incubated with primary antibodies against CD63 (1:1000 dilution; Abcam, Cambridge, UK), TSG101 (1:1000 dilution; Abcam), calnexin (1:500 dilution; Proteintech Group, Rosemont, IL, USA), SOCS3 (1:500 dilution; Proteintech Group), and β-tubulin (1:2000 dilution; Proteintech Group) at 4 °C overnight. The membranes were washed three times with 0.2% Tween 20 in PBS following incubation with alkaline phosphatase-conjugated secondary antibodies (SouthernBiotech, Birmingham, AL, USA).

### 4.9. Transmission Electron Microscopy

TEM was utilized to observe the exosome morphology. Exosome sample drops were allowed to adsorb to formvar-coated EM grids for 5 min, and were negatively stained with 2% (*w*/*v*) phosphotungstic acid for 1 min. TEM analysis was performed at an acceleration voltage of 80 kV with a transmission electron microscope (H-7650; Hitachi, Ltd., Tokyo, Japan).

### 4.10. Nanoparticle Tracking Analysis

Exosome sample concentration and size were detected using NTA with the NanoSight LM10 instrument (Malvern Panalytical, Malvern, UK) with a 488-nm laser and the accompanying NTA3.1 software. Five individual measurements of 30 s were recorded for each sample with automated analysis settings for blur, track length, and minimum expected particle size. The camera level was set at 14 and the detection threshold at 10.

### 4.11. Immunofluorescence Microscopy

Cells were fixed with 4% paraformaldehyde, permeabilized with 0.2% Triton X-100 for 15 min, and blocked with bovine serum albumin (Sigma-Aldrich Corporation, St. Louis, MO, USA). Cells were then washed three times with PBS and incubated with primary antibodies against fluorescein isothiocyanate-conjugated RABV N protein at room temperature (approximately 25 °C) for 2 h. Then, the cells were incubated with 4′,6-diamidino-2-phenylindole (KeyGen Biotech. Co., Ltd., Nanjing, China) at room temperature (approximately 25 °C) for 5 min. Cell fluorescence was visualized using the Zeiss LSM 710 (Carl Zeiss).

### 4.12. Statistical Analysis

Data represent at least three independent experiments and are presented as mean ± SEM. Data were analyzed using the GraphPad Prism software (GraphPad Software Inc., La Jolla, CA, USA). Student’s *t*-test was used for statistical analysis to compare the differences between treatment groups. A *p*-value < 0.05 was considered statistically significant.

## Figures and Tables

**Table 1:** List of primers used for quantitative reverse transcriptase PCR.

Gene	Forward Primer (5′-3′)	Reverse Primer (5′-3′)
*Rabies virus N protein*	CAAGATGTGTGCYAAYTGGAG	AGCCCTGGTTCGAACATTCT
*IFN-β*	GTCTCCTCCAAATTGCTCTC	ACAGGAGCTTCTGACACTGA
*STAT1*	TTCTGTGTCTGAAGTGTAAGTGAA	TAACACGGGGATCTCAACAAGTTC
*OAS1*	AGAAGGCAGCTCACGAAACC	CCACCACCCAAGTTTCCTGTA
*IRF7*	GAGCCCTTACCTCCCCTGTTAT	CCACTGCAGCCCCTCATAG
*18S*	CTTAGAGGGACAAGTGGCG	ACGCTGAGCCAGTCAGTGTA
miR-423-5p	GGGGTGAGGGGCAGAGAG	universal reverse primers

## Supplementary Materials

Supplementary materials can be found at https://www.mdpi.com/1422-0067/20/7/1537/s1. Table S1: Differentially expressed microRNAs between RABV-Exo and Mock-Exo samples. Figure S1: Western blot analysis of MRC-5 cells transfected with miR-383-5p inhibitor or miR-493-3p inhibitor. Figure S2: Rabies virus infection in MRC-5 cells and Vero cells.

## Abbreviations

DAPI	4′,6-diamidino-2-phenylindole
DiO	dioctadecyloxacarbocyanine perchlorate
Exo	exosome
HCV	hepatitis C virus
HDCs	human diploid cells
IFN	interferon
JAK-SATA	Janus Kinase-Signal Transducers and Activators of Transcription
KSHV	Kaposi’s sarcoma-associated herpesvirus
miRNA	microRNA
MRC-5 cells	human embryonic lung fibroblast cells
Mut	mutant
MVB	multivesicular body
NTA	nanoparticle tracking analysis
RABV	rabies virus
siRNA	small interfering RNA
SOCS3	suppressor of cytokine signaling 3
SSI	STAT-induced STAT inhibitor
TEM	transmission electron microscopy
WT	wild-type

## References

[1] Tordo N., Poch O., Ermine A., Keith G., Rougeon F. (1986). Walking along the rabies genome: Is the large G-L intergenic region a remnant gene?. Proc. Natl. Acad. Sci. USA.

[2] GBD 2015 Mortality and Causes of Death Collaborators (2016). Global, regional, and national life expectancy, all-cause mortality, and cause-specifi c mortality for 249 causes of death, 1980–2015: A systematic analysis for the Global Burden of Disease Study 2015. Lancet.

[3] Hicks D.J., Fooks A.R., Johnson N. (2012). Developments in rabies vaccines. Clin. Exp. Immunol..

[4] Kondo A. (1965). Growth characteristics of rabies virus in primary chicken embryo cells. Virology.

[5] Lavender J.F., Van Frank R.M. (1971). Zonal-centrifuged purifed duck embryo cell culture rabies vaccine for human vaccination. Appl. Microbiol..

[6] Majer M., Hilfenhaus A.J., Mauler R., Lehmann H.G., Hennessen W., Kuwert E.K. (1977). A comparison of the Pasteur and Pitman-Moore strains of rabies virus for the production of rabies vaccine in human diploid cells. J. Biol. Standard..

[7] Lin F., Lu L. (1983). Rabies vaccine production in animal cell cultures. J. Infect. Dis..

[8] Wu X., Smith T.G., Rupprecht C.E. (2011). From brain passage to cell adaptation: The road of human rabies vaccine development. Expert Rev. Vaccines.

[9] Jacobs J.P. (1970). Characteristics of a human diploid cell designated MRC-5. Nature.

[10] Maria I.T., Melitta S., Ursula H., Harold C., Monique L. (1998). The Neural Cell Adhesion Molecule Is a Receptor for Rabies Virus. J. Virol..

[11] Beutler B., Jiang Z., Georgel P., Crozat K., Croker B., Rutschmann S., Du X., Hoebe K. (2006). Genetic analysis of host resistance: Toll-like receptor signaling and immunity at large. Annu. Rev. Immunol..

[12] Kim B.H., Shenoy A.R., Kumar P., Bradfield C.J., MacMicking J.D. (2012). IFN-inducible GTPases in host cell defense. Cell Host Microbe.

[13] Assil S., Webster B., Dreux M. (2015). Regulation of the Host Antiviral State by Intercellular Communications. Viruses.

[14] Randall R.E., Goodbourn S. (2008). Interferons and viruses: An interplay between induction, signalling, antiviral responses and virus countermeasures. J. General Virol..

[15] Antwi-Baffour S.S. (2015). Molecular characterisation of plasma membrane-derived vesicles. J. Biomed. Sci..

[16] Tkach M., Thery C. (2016). Communication by Extracellular Vesicles: Where We Are and Where We Need to Go. Cell.

[17] Kalra H., Drummen G.P., Mathivanan S. (2016). Focus on Extracellular Vesicles: Introducing the Next Small Big Thing. Int. J. Mol. Sci..

[18] Schorey J.S., Cheng Y., Singh P.P., Smith V.L. (2015). Exosomes and other extracellular vesicles in host-pathogen interactions. EMBO Rep..

[19] Raab-Traub N., Dittmer D.P. (2017). Viral effects on the content and function of extracellular vesicles. Nat. Rev. Microbiol..

[20] Petrik J. (2016). Immunomodulatory effects of exosomes produced by virus-infected cells. Transf. Apher. Sci..

[21] Valadi H., Ekstrom K., Bossios A., Sjostrand M., Lee J.J., Lotvall J.O. (2007). Exosome-mediated transfer of mRNAs and microRNAs is a novel mechanism of genetic exchange between cells. Nat. Cell Biol..

[22] Ramakrishnaiah V., Thumann C., Fofana I., Habersetzer F., Pan Q., de Ruiter P.E., Willemsen R., Demmers J.A., Stalin Raj V., Jenster G. (2013). Exosome-mediated transmission of hepatitis C virus between human hepatoma Huh7.5 cells. Proc. Natl. Acad. Sci. USA.

[23] Lenassi M., Cagney G., Liao M., Vaupotic T., Bartholomeeusen K., Cheng Y., Krogan N.J., Plemenitas A., Peterlin B.M. (2010). HIV Nef is secreted in exosomes and triggers apoptosis in bystander CD4+ T cells. Traffic.

[24] Pleet M.L., DeMarino C., Lepene B., Aman M.J., Kashanchi F. (2017). The Role of Exosomal VP40 in Ebola Virus Disease. DNA Cell Biol..

[25] Yu X., Odenthal M., Fries J.W. (2016). Exosomes as miRNA Carriers: Formation-Function-Future. Int. J. Mol. Sci..

[26] Pegtel D.M., Cosmopoulos K., Thorley-Lawson D.A., van Eijndhoven M.A., Hopmans E.S., Lindenberg J.L., de Gruijl T.D., Wurdinger T., Middeldorp J.M. (2010). Functional delivery of viral miRNAs via exosomes. Proc. Natl. Acad. Sci. USA.

[27] Vojtech L., Woo S., Hughes S., Levy C., Ballweber L., Sauteraud R.P., Strobl J., Westerberg K., Gottardo R., Tewari M. (2014). Exosomes in human semen carry a distinctive repertoire of small non-coding RNAs with potential regulatory functions. Nucleic Acids Res..

[28] Kalamvoki M., Du T., Roizman B. (2014). Cells infected with herpes simplex virus 1 export to uninfected cells exosomes containing STING, viral mRNAs, and microRNAs. Proc. Natl. Acad. Sci. USA.

[29] Li J., Liu K., Liu Y., Xu Y., Zhang F., Yang H., Liu J., Pan T., Chen J., Wu M. (2013). Exosomes mediate the cell-to-cell transmission of IFN-alpha-induced antiviral activity. Nat. Immunol..

[30] Giugliano S., Kriss M., Golden-Mason L., Dobrinskikh E., Stone A.E.L., Soto-Gutierrez A., Mitchell A., Khetani S.R., Yamane D., Stoddard M. (2015). Hepatitis C Virus Infection Induces Autocrine Interferon Signaling by Human Liver Endothelial Cells and Release of Exosomes, Which Inhibits Viral Replication. Gastroenterology.

[31] Narayanan A., Jaworski E., Van Duyne R., Iordanskiy S., Guendel I., Das R., Currer R., Sampey G., Chung M., Kehn-Hall K. (2014). Exosomes derived from HTLV-1 infected cells contain the viral protein Tax. Retrovirology.

[32] Ariza M.E., Rivailler P., Glaser R., Chen M., Williams M.V. (2013). Epstein-Barr virus encoded dUTPase containing exosomes modulate innate and adaptive immune responses in human dendritic cells and peripheral blood mononuclear cells. PLoS ONE.

[33] Honegger A., Schilling D., Bastian S., Sponagel J., Kuryshev V., Sultmann H., Scheffner M., Hoppe-Seyler K., Hoppe-Seyler F. (2015). Dependence of intracellular and exosomal microRNAs on viral *E6/E7* oncogene expression in HPV-positive tumor cells. PLoS Pathog..

[34] Liu L., Zhou Q., Xie Y., Zuo L., Zhu F., Lu J. (2017). Extracellular vesicles: Novel vehicles in herpesvirus infection. Virol. Sin..

[35] Zhou Y., Wang X., Sun L., Zhou L., Ma T.C., Song L., Wu J.G., Li J.L., Ho W.Z. (2016). Toll-like receptor 3-activated macrophages confer anti-HCV activity to hepatocytes through exosomes. FASEB J..

[36] Kouwaki T., Okamoto M., Tsukamoto H., Fukushima Y., Oshiumi H. (2017). Extracellular Vesicles Deliver Host and Virus RNA and Regulate Innate Immune Response. Int. J. Mol. Sci..

[37] Bellingham S.A., Coleman B.M., Hill A.F. (2012). Small RNA deep sequencing reveals a distinct miRNA signature released in exosomes from prion-infected neuronal cells. Nucleic Acids Res..

[38] Verweij F.J., van Eijndhoven M.A., Middeldorp J., Pegtel D.M. (2013). Analysis of viral microRNA exchange via exosomes in vitro and in vivo. Methods Mol. Biol..

[39] Yogev O., Henderson S., Hayes M.J., Marelli S.S., Ofir-Birin Y., Regev-Rudzki N., Herrero J., Enver T. (2017). Herpesviruses shape tumour microenvironment through exosomal transfer of viral microRNAs. PLoS Pathog..

[40] Chugh P.E., Sin S.H., Ozgur S., Henry D.H., Menezes P., Griffith J., Eron J.J., Damania B., Dittmer D.P. (2013). Systemically circulating viral and tumor-derived microRNAs in KSHV-associated malignancies. PLoS Pathog..

[41] Song M.M., Shuai K. (1998). The suppressor of cytokine signaling (SOCS) 1 and SOCS3 but not SOCS2 proteins inhibit interferon-mediated antiviral and antiproliferative activities. J. Biol. Chem..

[42] Kreuzer R.S., Bekurtz J.C., Arends D., Bortfeldt R., Kutz L.B., Sharbati S., Einspanier R., Brockmann G.A. (2016). Feeding of Enterococcus faecium NCIMB 10415 Leads to Intestinal miRNA-423-5p-Induced Regulation of Immune-Relevant Genes. Appl. Environ. Microbiol..

[43] Wang W.G., Wang F. (2017). MiR-663a/MiR-423-5p are involved in the pathogenesis of lupus nephritis via modulating the activation of NF-κB by targeting TNIP2. Am. J. Transl. Res..

[44] Théry C., Amigorena S., Raposo G., Clayton A. (2006). Isolation and Characterization of Exosomes from Cell Culture Supernatants and Biological Fluids.

[45] Liang G., Kan S., Zhu Y., Feng S., Feng W., Gao S. (2018). Engineered exosome-mediated delivery of functionally active miR-26a and its enhanced suppression effect in HepG2 cells. Int. J. Nanomed..

[46] Wang L., Liu Y., Zhang S., Wang Y., Zhao J., Miao F., Hu R. (2014). A SYBR-green I quantitative real-time reverse transcription-PCR assay for rabies viruses with different virulence. Virol. Sin..

[47] Remoli M.E., Giacomini E., Lutfalla G., Dondi E., Orefici G., Battistini A., Uze G., Pellegrini S., Coccia E.M. (2002). Selective Expression of *Type I IFN* Genes in Human Dendritic Cells Infected with Mycobacterium tuberculosis. J. Immunol..

