# Supplementary figures and images for: Exosome-Mediated Delivery of Inducible miR-423-5p Enhances Resistance of MRC-5 Cells to Rabies Virus Infection
Source: Int J Mol Sci. 2019 Mar 27;20(7):1537. doi: 10.3390/ijms20071537 (PMC6479321; doi:10.3390/ijms20071537)

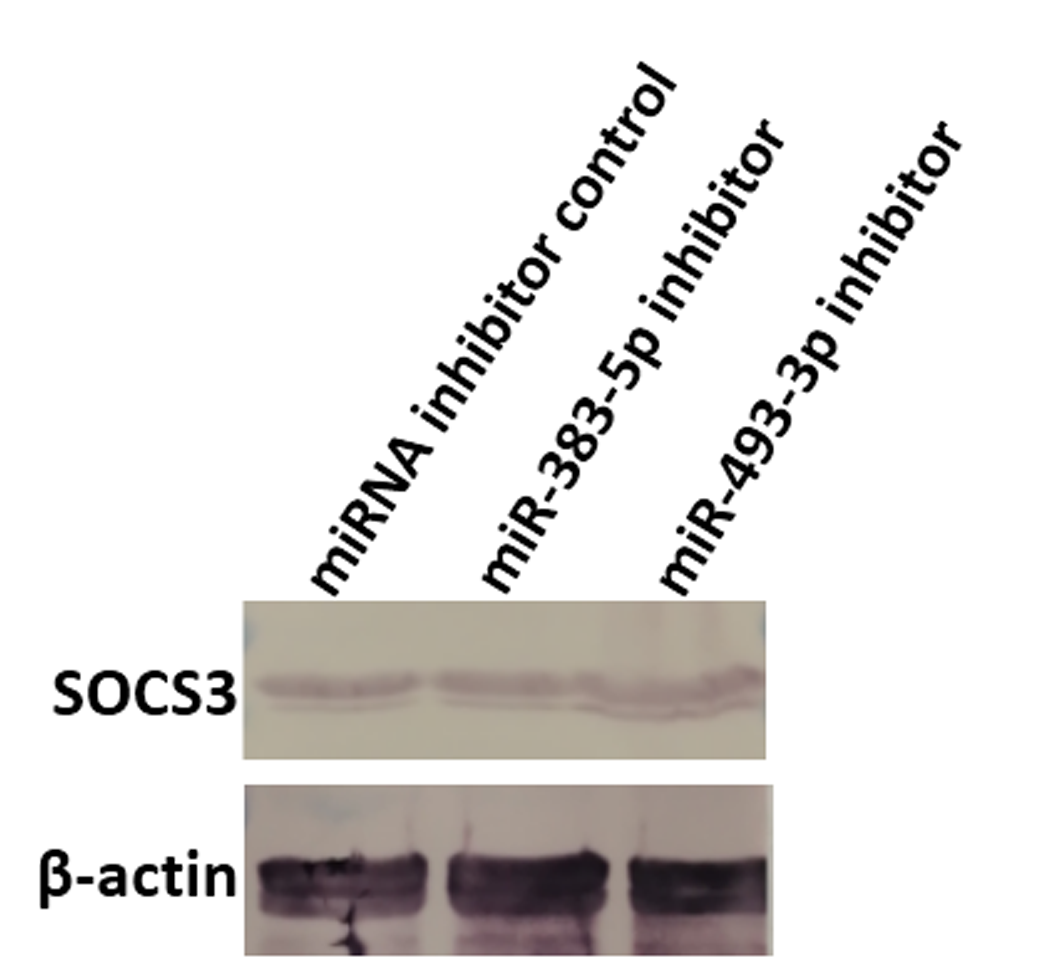

Supplement: Supplementary file 1 [file ijms-20-01537-s001.zip › Figure S1.tif]

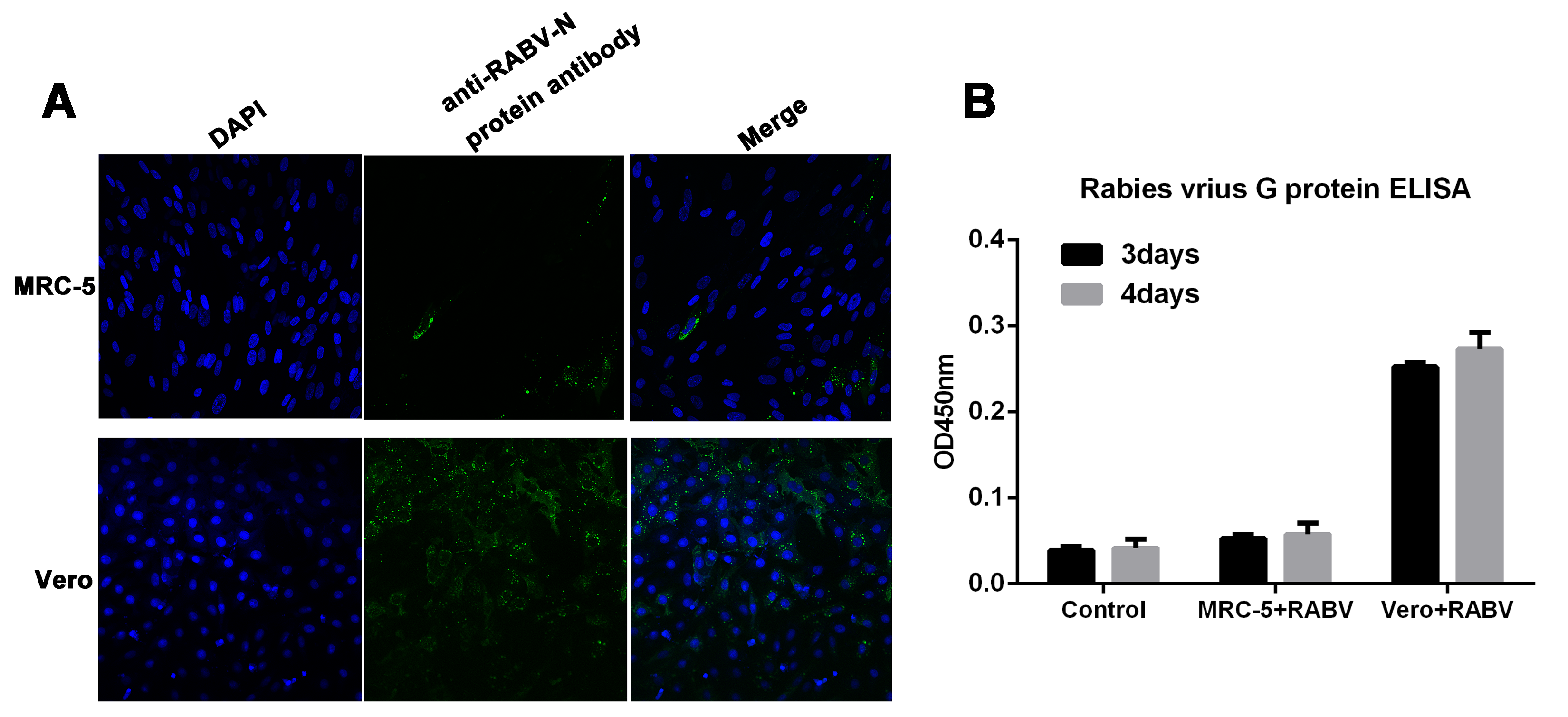

Supplement: Supplementary file 1 [file ijms-20-01537-s001.zip › Figure S2.tif]
